# Chemistry of Renieramycins. Part 14: Total Synthesis of Renieramycin I and Practical Synthesis of Cribrostatin 4 (Renieramycin H)

**DOI:** 10.3390/md13084915

**Published:** 2015-08-06

**Authors:** Masashi Yokoya, Keiichiro Kobayashi, Mitsuhiro Sato, Naoki Saito

**Affiliations:** Graduate School of Pharmaceutical Sciences, Meiji Pharmaceutical University, 2-522-1 Noshio, Kiyose, Tokyo 204-8588, Japan; E-Mails: yokoya@my-pharm.ac.jp (M.Y.); k.koba0222@gmail.com (K.K.); m146212@std.my-pharm.ac.jp (M.S.)

**Keywords:** cribrostatin 4 (renieramycin H), renieramycin I, total synthesis, structural determination, selenium oxide oxidation, marine natural product

## Abstract

The first total synthesis of (±)-renieramycin I, which was isolated from the Indian bright blue sponge *Haliclona cribricutis*, is described. The key step is the selenium oxide oxidation of pentacyclic bis-*p*-quinone derivative (**3**) stereo- and regioselectively. We also report a large-scale synthesis of cribrostatin 4 (renieramycin H) via the C3-C4 double bond formation in an early stage based on the Avendaño’s protocol, from readily available 1-acetyl-3-(3-methyl-2,4,5-trimethylphenyl)methyl-piperazine-2,5-dione (**8**) in 18 steps (8.3% overall yield). The synthesis provides unambiguous evidence supporting the original structure of renieramycin I.

## 1. Introduction

**Figure 1:**
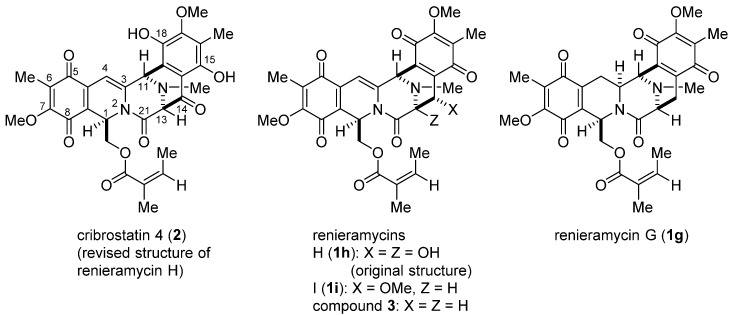
Structures of bis-1,2,3,4-tetrahydroisoquinoline marine natural products.

Many tetrahydroisoquinoline antitumor natural products, such as renieramycins, saframycins, and ecteinascidins, have attracted considerable interest due to their extraordinary structures and meager availability in nature, as well as their potent antitumor activity [1,2]. Among them, renieramycins H (**1h**) and I (**1i**) were isolated from the methanol extract of the Indian bright blue sponge *Haliclona cribricutis* collected from the intertidal region of Okha, Gujarat, in 1988 [3]. Original structures **1h** and **1i** were given the names renieramycins H and I, respectively. Thereafter, we revised the structure of renieramycin H to that of cribrostatin 4 (**2**) [4,5,6], which was independently isolated from the blue sponge *Cribrochalina* sp. collected from reef passages in the Republic of Maldives, based on ^13^C NMR studies of several semi-synthetic models (Figure 1) [7,8]. Cribrostatin 4 (**2**) has attracted the interest of several medicinal chemistry experts because of its unique structure and cytotoxicity despite the lack of the hemiaminal or aminonitrile function at C-21. Three total syntheses of **2** have been reported [9,10,11]. Recently, we completed a 21-step stereocontrolled total synthesis of (±)**-2** from 1-acetyl-3-(3-methyl-2,4,5-trimethylphenyl)methyl-piperazine-2,5-dione (**8**) in 3.4% overall yield [12,13]. Furthermore, we have accomplished the total synthesis of renieramycin G (**1g**) [14,15]. The availability of **1g** and **2** has enabled us to prepare several renieramycin derivatives having a lactam carbonyl to understand the molecular basis of their impressive cytotoxicity profiles. We present herein an alternative large-scale approach for the total synthesis of **2**. This approach might yield a variety of novel analogs of cribrostatin 4 (**2**), as well as C3-C4 unsaturated bis-*p*-quinone derivatives, such as renieramycin I (**1i**), for detailed studies of structure activity relationships (SARs) of these classes of antitumor marine natural products. 

## 2. Results 

**Chart 1:**
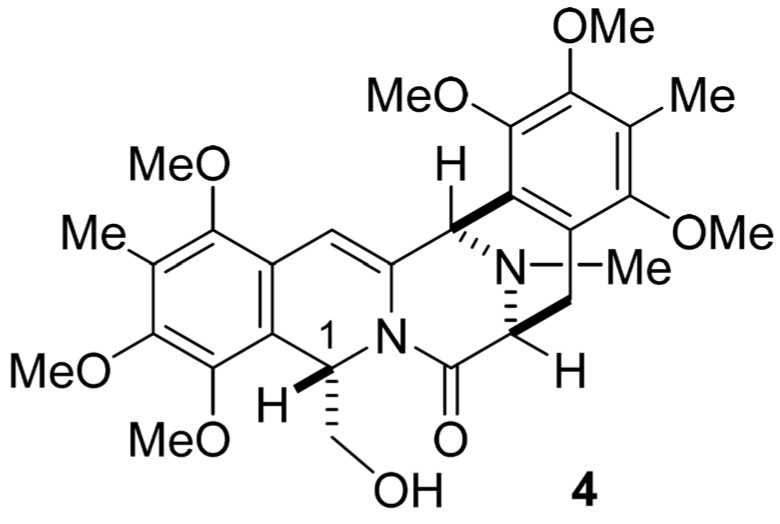
Structure of 1-*epi*-pentacyclic alcohol.

**Scheme 1:**
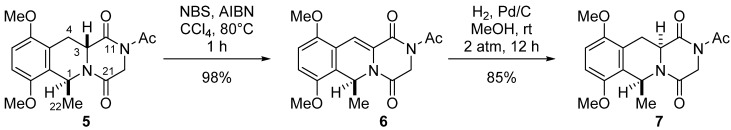
Epimerization at C-3 through regioselective bromination at C-3 position and reduction sequences by Avendaño and co-workers.

The most serious problem in our previous cribrostatin 4 (**2**) synthesis was that 1-*epi*-pentacyclic alcohol (**4**) (Chart 1) might be formed, and the undesired stereochemistry had to be converted into the natural one at C-1 position via enolate formation through several cycles. Avendaño *et al*. reported that the stereocenter at C-3 of 1,3-*trans*-compound **5** [16] could be transformed into corresponding 1,3-*cis*-compound **7** via unsaturated compound **6** through regioselective radical bromination, followed by hydrogenation from the less hindered α-face in good yield [17]. They applied this protocol to the preparation of pentacyclic phthalascidin analogs [18]. We were very interested in this procedure for constructing the 1,3-*cis* relationships of renieramycins (Scheme 1) [19]. 

**Scheme 2:**
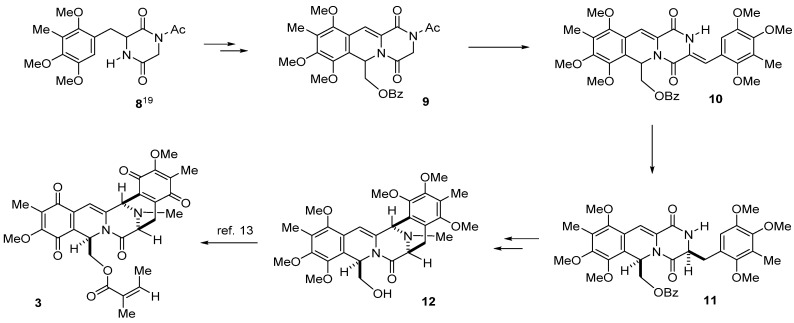
Strategy for practical synthesis of compound **3**, which will be converted into **1i** and **2**.

Based on Avendaño’s protocol, we designed an alternative synthetic plan that involves the key transformations outlined in Scheme 2: (1) construction of tricyclic compound **9** having an α,β-unsaturated amide carbonyl from readily available compound **8** [20]; (2) condensation of **9** with benzaldehyde derivative and subsequent regio- and stereospecific hydrogenation leading to compound **11**; (3) construction of pentacyclic framework and conversion of ester into our intermediate **12**, which can be transformed into cribrostatin 4 intermediate **3** [13] (Scheme 2).

**Scheme 3:**
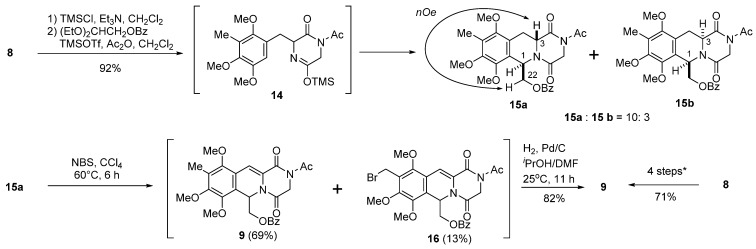
**8** → **9**: (1) TMSCl, TEA, CH_2_Cl_2_; (2) (EtO)_2_CHCH_2_OBz, TMSOTf, Ac_2_O, CH_2_Cl_2_; (3) NBS, CCl_4_, 60 °C, 3 h; (4) H_2_, Pd/C, *^i^*PrOH/DMF, 25 °C, 11 h.

According to the results of our previous studies [21,22], treatment of **8** with trimethylsilyl chloride (TMSCl) in the presence of triethylamine (TEA) in CH_2_Cl_2_ gave *O*-trimethylsilyl lactim intermediate **14**, which was treated with 2,2-diethoxyethyl benzoate in the presence of trimethylsilyl triflate (TMSOTf) and acetic anhydride to give **15** as an inseparable mixture of diastereomers (**15a**:**15b** = 10:3) in 92% yield. After exerting a great deal of effort to separate this mixture by column chromatography several times, we obtained both isomers in their pure forms, and detailed 2D NMR studies confirmed the structures of **15a** (minor) and **15b** (major). The NMR spectrum of **15a** displayed H-1 and H-3 proton signals at δ 6.20 and δ 4.70, respectively, whereas the NMR spectrum of **15b** showed H-1 and H-3 proton signals appearing at δ 6.15 and δ 4.07, respectively. An observable nuclear Overhauser enhancement (NOE) between H-3 and H-22 revealed that compound **15a** has the *trans* form (Scheme 3). 

We then studied the conversion of **15** into unsaturated compound **9**, which is the first key step of our synthesis. A preliminary experiment was carried out using major isomer **15a**. According to the typical conditions of Avendaño *et al*. [17], **15a** was treated with *N*-bromosuccinimide (NBS: 1.0 equiv.) and 2,2′-azobisisobutyronitrile (AIBN: 0.1 equiv.) in CCl_4_ at 80 °C for 6 h to generate **9** (55%) and **16** (4%) plus unreacted **15a** (33%). The ^1^H NMR spectrum of **9** showed an H-4 olefinic proton signal that appeared as a singlet at δ 7.47. The ^1^H NMR spectrum of **16** showed characteristic AB type doublet proton signals at δ 4.60 and δ 4.57 along with the H-4 olefinic singlet proton signal at δ 7.41. Accordingly, **16** might be a product of over-reaction product at C-6 aromatic methyl group. After extensive investigation of the reaction conditions, we found that the yield of our target **9** could be improved by slightly lowering the reaction temperature (60 °C) and excluding AIBN. Thus, the reaction of **15a** with NBS (2 equiv.) in CCl_4_ at 60 °C for 6 h gave **9** (69%) and **16** (13%). It was extremely difficult to separate **9** and **16** in a large scale using silica gel column chromatography. However, catalytic reduction of the above mixture using 10% Pd/C in 2-propanol and DMF at 25 °C for 11 h gave **9** as the sole product in 82% overall yield. Accordingly, the transformation of **15a** into **9** without any purification of the intermediates was found to be the best choice in terms of overall yield (**9** in 71% yield in four steps). 

**Scheme 4:**
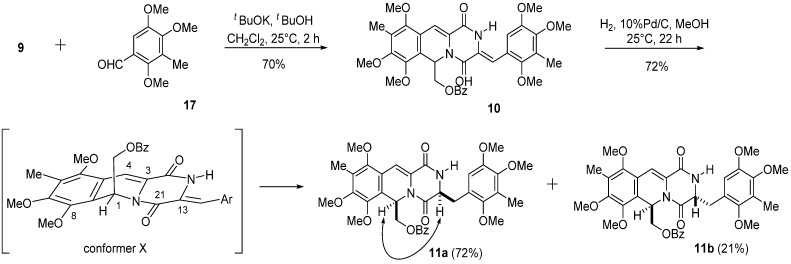
Preparation of key intermediate **11a**.

**Scheme 5:**
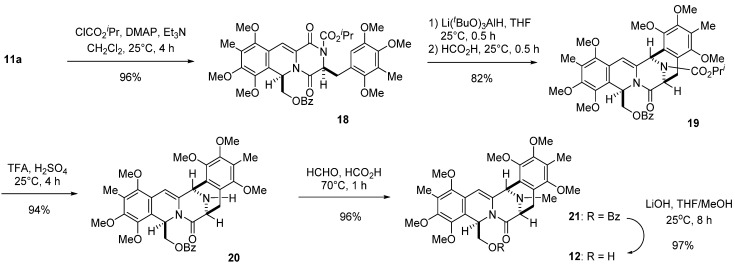
Construction of pentacyclic primary alcohol **12**.

With key intermediate **9** in hand, we next looked into ways to design a practical transformation of **9** into **12**, which was the key intermediate in our previous total synthesis of cribrostatin 4 (**2**) (Scheme 4 and Scheme 5). Condensation of **9** with benzaldehyde derivative **17** [20] in the presence of potassium *tert*-butoxide gave (*Z*)-arylidenepiperazinedione **10** in 70% yield. Catalytic hydrogenation of the trisubstituted double bond of **10** over 10% Pd on carbon in MeOH at 25 °C proceeded chemoselectively to give desired **11a** (72%) along with **11b** (21%). Detailed 2D NMR studies were performed to confirm the structures of **11a** and **11b**. The NMR spectrum of **11a** displayed H-1 and H-13 proton signals at δ 6.53 and δ 4.34, respectively, whereas the NMR spectrum of **11b** had H-1 and H-13 proton signals appearing δ 6.43 and δ 4.45, respectively. An NOE between H-1 and H-13 proton signals was observed in **11a** but not **11b**. Thus, the hydrogenation of **10** obviously occurred stereoselectively from the α-face to generate H-1 and H-13 cis isomer **11a**. It is proposed that the steric hindrance due to the C-8 methoxy group and the C-21 carbonyl group was responsible for the β-axial orientation of C-1 substituent as shown in conformer X of **10**. 

The piperazinedione ring of **11a** was activated by introducing a 2-propyloxycarbonyl group to give imide **18** in 96% yield. Chemoselective reduction of **18** in the conventional manner afforded a hemiaminal, which was treated with formic acid at 25 °C for 0.5 h to afford **19** [23] in 82% yield. Deprotection of **19** with TFA and H_2_SO_4_ gave secondary amine **20**, which was transformed into **21** by reductive methylation in high yield. Hydrolysis of **21** with 10 N aqueous LiOH in THF/MeOH at 25 °C for 8 h gave primary alcohol **12** in 97% yield, which is identical to the intermediate in our previous total synthesis [13].

**Scheme 6:**
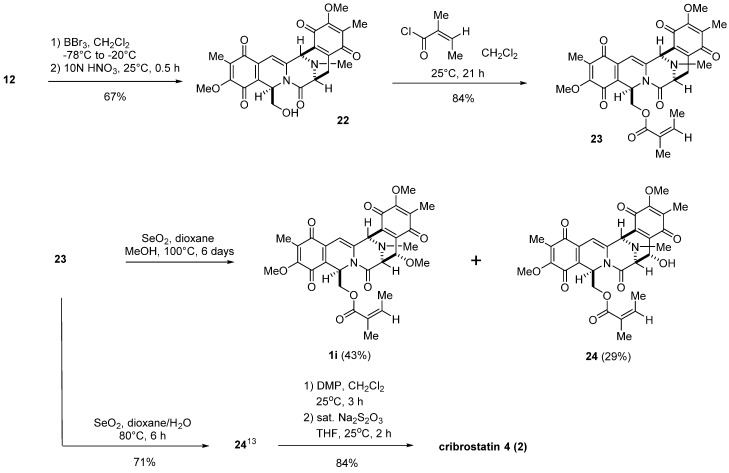
Transformation of compound **12** into renieramycin I (**1i**) and cribrostatin 4 (**2**) through compound **23**.

The conversion of **12** into **22** was accomplished by partial demethylation with boron tribromide (BBr_3_), followed by oxidative demethylation to give bis-*p*-quinone **22** in 67% yield (Scheme 6). Acylation of **22** with in situ prepared angeloyl chloride in dichloromethane gave common intermediate **23** in 84% yield. Encouraged by the results of our extensive model studies, including the transformation of several natural products [24,25,26], the introduction of a methoxy group to the C-14 position of **23** was achieved using 10 equiv. of SeO_2_ in a mixture of methanol and dioxane at 100 °C for six days to give **1i** in 43% yield along with secondary alcohol **24** in 29% yield. The orientation of the methoxy group of **1i** was assigned on the basis of the signal of 14-H (δ 4.34, d, *J* = 1.4 Hz). The spectroscopic properties of synthetic **1i** were in complete accord with those of natural renieramycin I (**1i**) [27].

## 3. Experimental Section 

IR spectra were obtained with a Shimadzu Prestige 21/IRAffinity-1 FT-IR spectrometer. ^1^H- and ^13^C-NMR spectra were recorded on a JEOL JNM-ECA 500 FT NMR spectrometer at 500 MHz for ^1^H and 125 MHz for ^13^C; a JEOL JNM-AL 400 NMR spectrometer at 400 MHz for ^1^H and 100 MHz for ^13^C; and a JEOL JNM-AL 300 NMR spectrometer at 300 MHz for ^1^H and 75 MHz for ^13^C (ppm, *J* in Hz with TMS as internal standard). All proton and carbon signals were assigned by extensive NMR measurements using COSY, HMBC, and HMQC techniques. Mass spectra were recorded on a JEOL JMS 700 instrument with a direct inlet system operating at 70 eV. Elemental analyses were conducted on a YANACO MT-6 CHN CORDER elemental analyzer.

### 3.1. ((6R*,11aR*)-2-Acetyl-7,8,10-trimethoxy-9-methyl-1,4-dioxo-1,3,4,6,11,11a-hexahydro-2H-pyrazino-(1,2-b)isoquinolin-6-yl)methyl Benzoate (**15a**) and ((6R*,11aS*)-2-acetyl-7,8,10-trimethoxy-9-methyl- 1,4-dioxo-1,3,4,6,11,11a-hexahydro-2H-pyrazino(1,2-b)isoquinolin-6-yl)methyl Benzoate (**15b**)

TMSCl (498 μL, 3.9 mmol) was added to a stirred solution of **8** (1.05 g, 3.0 mmol) in dichloromethane (18 mL) and TEA (544 μL, 3.9 mmol), and stirring was continued at 25 °C for 2 h. A solution of 2,2-diethoxyethyl benzoate [28] (785.8 mg, 3.3 mmol) in dichloromethane (12 mL) followed by TMSOTf (2.71 mL, 15 mmol) was added dropwise for 5 min each, and then Ac_2_O (283.6 μL, 3.0 mmol) was added in one portion at 25 °C and the reaction mixture was stirred for 4 h. The reaction mixture was diluted with saturated NaHCO_3_ solution (100 mL) and extracted with CHCl_3_ (100 mL × 3). The combined extracts were washed with brine (100 mL), dried, and concentrated *in vacuo*. The residue was subjected to column chromatography with ethyl acetate–hexane (1:2) to give **15** (1.37 g, 92%, **15a**:**15b** = 10:3) as a colorless amorphous powder, which was an inseparable mixture of diastereomers. Each authentic sample was obtained by chromatography on preparative layer silica gel plates (Merck 5715).

Compound **15a**: ^1^H-NMR (400 MHz, CDCl_3_) δ: 7.99 (2H, m, Ar-H), 7.57 (1H, m, Ar-H), 7.44 (2H, m, Ar-H), 6.20 (1H, dd, *J* = 9.5, 3.9 Hz, C6-H), 4.75 (1H, dd, *J* = 11.7, 9.5 Hz, C12-H), 4.70 (1H, dd, *J* = 9.8, 5.4 Hz, C11a-H), 4.52 (1H, dd, *J* = 11.7, 3.9 Hz, C12-H), 4.36 (1H, d, *J* = 18.0 Hz, C3-H), 4.31 (1H, d, *J* = 18.0, C3-H), 3.95 (3H, s, C7-OMe), 3.80 (3H, s, C8-OMe), 3.72 (3H, s, C10-OMe), 3.40 (1H, dd, *J* = 16.6, 5.4 Hz, C11-Hα), 3.14 (1H, dd, *J* = 16.6, 9.8 Hz, C11-Hβ), 2.59 (3H, s, COCH_3_), 2.21 (3H, s, C9-Me). ^13^C-NMR (100 MHz, CDCl_3_) δ: 171.7 (s, COCH_3_), 167.9 (s, C1), 166.6 (s, OCOPh), 163.2 (s, C4), 152.4 (s, C10), 150.6 (s, C8), 146.2 (s, C7), 133.3 (d, Ph), 129.7 (d, Ph × 2), 129.6 (s, Ph), 128.5 (d, Ph × 2), 125.9 (s, C9), 122.6 (s, C6a), 121.4 (s, C10a), 63.6 (t, C12), 60.6 (q, C7-OCH_3_), 60.3 (q, C10-OCH_3_), 60.0 (q, C8-OCH_3_), 54.0 (d, C11a), 48.3 (d, C6), 45.7 (t, C3), 27.2 (q, COCH_3_), 26.0 (t, C11), 9.5 (q, C9-CH_3_). FT-IR (KBr) cm^−1^: 1717, 1686, 1672, 1275, 714. EI-MS *m/z* (%): 496 (M^+^, 11), 361 (100), 319 (13), 234 (15), 204 (8), 105 (7). HR-EI-MS: calcd for C_26_H_28_N_2_O_8_, 496.1846, found: 496.1841.

Compound **15b**: ^1^H-NMR (400 MHz, CDCl_3_) δ: 7.93 (2H, m, Ar-H), 7.54 (1H, m, Ar-H), 7.42 (2H, m, Ar-H), 6.15 (1H, dd, *J* = 7.0, 4.6 Hz, C6-H), 5.10 (1H, d, *J* = 16.0 Hz, C3-H), 4.55 (1H, dd, *J* = 11.5, 7.0 Hz, C12-H), 4.37 (1H, dd, *J* = 11.5, 4.6 Hz, C12-H), 4.07 (1H, dd, *J* = 12.2, 4.9 Hz, C11a-H), 3.94 (3H, s, C7-OMe), 3.79 (3H, s, C8-OMe), 3.78 (1H, d, *J* =16.0 Hz, C3-H), 3.68 (1H, dd, *J* =15.9, 4.9 Hz, C11-Hα), 3.64 (3H, s, C10-OMe), 3.05 (1H, dd, *J* = 15.9, 12.2 Hz, C11-Hβ), 2.59 (3H, s, COMe), 2.22 (3H, s, C9-Me). ^13^C-NMR (100 MHz, CDCl_3_) δ: 170.9 (s, COCH_3_), 168.7 (s, C1), 166.4 (s, OCOPh), 166.1 (s, C4), 151.7 (s, C10), 150.6 (s, C8), 146.2 (s, C7), 133.2 (d, Ph × 2), 129.7 (s, Ph), 129.6 (d, Ph), 128.4 (d, Ph × 2), 126.1 (s, C9), 123.6 (s, C6a), 121.8 (s, C10a), 66.1 (t, C12), 61.0 (q, C10-OCH_3_), 60.7 (q, C7-OCH_3_), 60.0 (q, C8-OCH_3_), 56.3 (d, C11a), 48.5 (d, C6), 45.6 (t, C3), 26.9 (q, COCH_3_), 22.8 (t, C11), 9.4 (q, C9-CH_3_). FT-IR (KBr) cm^−1^: 1721, 1707, 1692, 1412, 1368, 1273. EI-MS *m/z* (%): 496 (M^+^, 10), 361 (100), 319 (11), 234 (13), 204 (7), 105 (7). HR-EI-MS: calcd for C_26_H_28_N_2_O_8_, 496.1846, found: 496.1841.

### 3.2. (2-Acetyl-7,8,10-trimethyl-1,4-dioxo-1,3,4,6-tetrahydro-2H-pyrazino(1,2-b)Isoquinoline-6-yl)methyl Beozoate (**9**) and (2-acetyl-9-(bromomethyl)-,7,8,10-trimethoxy-1,4-dioxo-1,3,4,6-tetrahydro-2H-pyrazino(1,2-b)isoquinolin-6-yl)methyl Beozoate (**16**)

NBS (106.8 mg, 0.6 mmol) was added to a stirred solution of **15a** (150.0 mg, 0.3 mmol) in dry CCl_4_, and the reaction mixture was heated at 60 °C for 6 h. The reaction mixture was filtered through a short pad of Celite, and the filtrate was washed with CCl_4_. The combined filtrates were concentrated *in vacuo*, and the residue was purified by flash chromatography on silica gel with ethyl acetate–hexane (1:3) as solvent to give **9** (102.0 mg, 69%) and **16** (17.7 mg, 13%).

Compound **9**: ^1^H-NMR (400 MHz, CDCl_3_) δ: 7.91 (2H, m, Ar-H), 7.55 (1H, m, Ar-H), 7.47 (1H, s, C11-H), 7.42 (2H, m, Ar-H), 6.47 (1H, dd, *J* = 7.7, 3.8 Hz, C6-H), 4.93 (1H, d, *J* = 17.6 Hz, C3-H), 4.52 (1H, dd, *J* = 11.7, 7.7 Hz, C12-H), 4.27 (1H, dd, *J* = 11.7, 3.8 Hz, C12-H), 3.96 (3H, s, C7-OMe), 3.92 (1H, d, *J* =17.6 Hz, C3-H), 3.85 (3H, s, C8-OMe), 3.71 (3H, s, C10-OMe), 2.61 (3H, s, COMe), 2.21 (3H, s, C9-Me). ^13^C-NMR (100 MHz, CDCl_3_) δ: 171.8 (s, COCH_3_), 166.3 (s, OCOPh), 162.7 (s, C4), 160.7 (s, C1), 154.1 (s, C8), 152.4 (s, C10), 146.0 (s, C7), 133.3 (d, Ph), 129.6 (d, Ph × 2), 129.4 (s, Ph), 128.5 (d, Ph × 2), 126.7 (s, C9), 125.8 (s, C11a or C10a), 121.5 (s, C6a), 118.9 (s, C11a or C10a), 114.7 (d, C11), 64.3 (t, C12), 62.1 (q, C10-OCH_3_), 60.8 (q, C7-OCH_3_), 60.2 (q, C8-OCH_3_), 47.9 (d, C6), 45.2 (t, C3), 26.7 (q, COCH_3_), 9.4 (q, C9-CH_3_). FT-IR (KBr) cm^−1^: 1701, 1416, 1393, 1368, 1269, 1211, 1088, 1072, 1043, 1028, 1007, 964, 459. EI-MS *m*/*z* (%): 494 (M^+^, 14), 359 (100), 260 (84), 232 (38), 217 (6), 202 (5), 105 (6). HR-EI-MS: calcd for C_26_H_26_N_2_O_8_, 494.1689, found: 494.1686.

Compound **16**: ^1^H-NMR (400 MHz, CDCl_3_) δ: 7.90 (2H, m, Ar-H), 7.55 (1H, m, Ar-H), 7.42 (2H, m, Ar-H), 7.41 (1H, s, C11-H), 6.49 (1H, dd, *J* = 7.7 Hz, 3.8 Hz, C6-H), 4.94 (1H, d, *J* =17.6 Hz, C3-H), 4.60 (1H, d, *J* =9.1 Hz, C9-CH_2_Br), 4.57 (1H, d, *J* = 9.1, C9-CH_2_Br), 4.55 (1H, dd, *J* = 11.6 Hz, 7.7 Hz, C12-H), 4.30 (1H, dd, *J* = 11.6 Hz, 3.8 Hz, C12-H), 4.03 (3H, s, C8-OMe), 3.97 (3H, s, C7-OMe), 3.90~3.95 (1H, overlapped, C3-H), 3.90 (3H, s, C10-OMe), 2.62 (3H, s, COCH_3_). ^13^C-NMR (100 MHz, CDCl_3_) δ: 171.8 (s, COCH_3_), 166.3 (s, OCOPh), 162.7 (s, C4), 160.4 (s, C1), 153.9 (s, C8), 152.1 (s, C10), 146.0 (s, C7), 133.4 (d, Ph), 129.6 (d, Ph × 2), 129.3 (s, Ph), 128.5 (d, Ph × 2), 127.3 (s, C9), 126.3 (s, C10a or C11a), 125.1 (s, C6a), 119.4 (s, C10a or C11a), 113.6 (d, C11), 64.2 (t, C12), 63.4 (q, C10-OCH_3_), 60.7 (q, C7-OCH_3_ and C8-OCH_3_), 47.9 (d, C6), 45.2 (t, C3), 26.7 (q, COCH_3_), 21.5 (t, C9-CH_2_Br). FT-IR (KBr) cm^−1^: 1701, 1368, 1292, 1269, 1215. EI-MS *m*/*z* (%): 572 (M^+^, 15), 493 (9), 439 (100), 417 (14), 393 (13), 375 (5), 371 (8), 364 (8), 359 (24), 339 (11), 310 (21), 274 (9), 260 (27), 231 (10), 105 (21). HR-EI-MS: calcd for C_26_H_25_BrN_2_O_8_, 572.0794, found: 572.0796.

### 3.3. (2-Acetyl-7,8-10-trimethyl-1,4-dioxo-1,3,4,6-tetrahydro-2H-pyrazino(1,2-b)isoquinolin-6-yl)methyl Benzoate (**9**) from **8** in Four Steps

TMSCl (498 μL, 3.9 mmol) was added to a stirred solution of **8** (1.05 g, 3.0 mmol) in dichloromethane (18 mL) and TEA (544 μL, 3.9 mmol), and stirring was continued at 25 °C for 2 h. A solution of 2,2-diethoxyethyl benzoate (785.8 mg, 3.3 mmol) in dichloromethane (12 mL) followed by TMSOTf (2.71 mL, 15 mmol) was added dropwise respectively over 5 min. Then, Ac_2_O (283.6 μL, 3.0 mmol) was added at 25 °C and the reaction mixture was stirred for 4 h. The reaction mixture was diluted with saturated NaHCO_3_ solution (100 mL) and extracted with CHCl_3_ (100 mL × 3). The combined extracts were washed with brine (100 mL), dried, and concentrated *in vacuo*. The residue was subjected to column chromatography with ethyl acetate–hexane (1:2) to give **15** (1.37 g, 92%, **15a**:**15b** = 10:3) as a colorless amorphous powder. Diastereomeric mixture **15** was dissolved in CCl_4_ (90 mL) and NBS (106.8 mg, 5.46 nmol) was added at 25 °C, and the reaction mixture was heated at 60 °C for 3 h. The reaction mixture was filtered through a short pad of Celite, and the filtrate was washed with CCl_4_. The combined filtrates were concentrated *in vacuo*, and the residue was used in the next step without further purification. The above residue was dissolved in 2-propanol/ DMF (1:0.2) (72 mL) and was hydrogenated over 10% Pd/C (980 mg) at 25 °C for 11 h. The catalyst was removed by filtration and washed with CHCl_3_ and MeOH. The combined filtrates were diluted with H_2_O (100 mL) and extracted with CHCl_3_ (100 mL × 3). The combined extracts were washed with brine (100 mL), dried, and concentrated *in vacuo*. The residue was subjected to column chromatography with ethyl acetate–hexane (1:4) to give **9** (1.05 g, 71% overall yield, 4 steps) as a colorless amorphous powder. 

### 3.4. (Z)-(7,8,10-Trimethoxy-9-methyl-1,4-dioxo-3-(2,4,5-trimethoxy-3-methylbenzylidene)-1,3,4,6-tetra-hydro-2H-pyrazino(1,2-b)isoquinolin-6-yl)methyl Benzoate (**10**)

A solution of *^t-^*BuOK in *^t-^*BuOH (1 M, 2.4 mL, 2.4 mmol) was added to a solution of **9** (988 mg, 2.0 mmol) and 2,4,5-trimethoxybenzaldehyde (**17**) (420 mg, 2.0 mmol) in CH_2_Cl_2_ (20 mL) over 1 h at 0 °C, and the reaction mixture was stirred at 25 °C for 2 h. The reaction mixture was diluted with saturated NH_4_Cl (100 mL) and extracted with CH_2_Cl_2_ (100 mL × 3). The combined extracts were washed with brine (100 mL), dried, and concentrated *in vacuo*. The residue was subjected to column chromatography with ethyl acetate–hexane (1:2) to give **10** (901 mg, 70%) as a pale yellow amorphous powder.

Compound **10**: ^1^H-NMR (400 MHz, CDCl_3_) δ: 9.40 (1H, s, N-H), 7.93 (2H, m, Ar-H), 7.49 (1H, m, Ar-H), 7.36 (2H, m, Ar-H), 7.31 (1H, s, C11-H), 6.94 (1H, s, C3a-H), 6.68 (1H, dd, *J* = 6.8, 3.9 Hz, C6-H), 6.62 (1H, s, C6′-H), 4.52 (1H, dd, *J* = 11.7, 6.8 Hz, C12-H), 4.45 (1H, dd, *J* = 11.7, 3.9 Hz, C12-H), 3.98 (3H, s, C7-OMe), 3.84 (3H, s, C8-OMe), 3.83 (3H, s, C4′-OMe), 3.83 (3H, s, C5′-OMe), 3.69 (3H, s, C10-OMe), 3.58 (3H, s, C2′-OMe), 2.24 (3H, s, C3′-Me), 2.19 (3H, s, C9-Me). ^13^C-NMR (100 MHz, CDCl_3_) δ: 166.1 (s, OCOPh), 157.4 (s, C4), 156.5 (s, C1), 153.3 (s, C8), 151.8 (s, C10), 149.6 (s, C5′), 149.2 (s, C2′), 149.0 (s, C4′), 146.0 (s, C7), 132.9 (d, Ph), 129.7 (s, Ph), 129.7 (d, Ph × 2), 128.2 (d, Ph × 2), 126.6 (s, C3′), 126.4 (s, C9), 126.2 (s, C11a), 125.4 (s, C3), 121.6 (s, C1′), 120.6 (s, C6a), 119.1 (s, C10a), 114.0 (d, C3a), 111.9 (d, C6′), 110.8 (d, C11), 65.0 (t, C12), 62.0 (q, C10-OCH_3_), 60.9 (q, C2′-OCH_3_), 60.7 (q, C7-OCH_3_), 60.4 (q, C4′-OCH_3_), 60.1 (q, C8-OCH_3_), 55.9 (q, C5′-OCH_3_), 48.7 (d, C6), 9.5 (q, C3′-CH_3_), 9.3 (q, C9-CH_3_). FT-IR (KBr) cm^−1^: 3246, 1724, 1688, 1628, 1489, 1468, 1452, 1414, 1387, 1369, 1323, 1269, 1248, 1090, 1067, 1003, 712. EI-MS *m/z* (%): 644 (M^+^, 7), 509 (100), 481 (5), 232 (10). HR-EI-MS: calcd for C_35_H_36_N_2_O_10_, 644.2370, found: 644.2369. 

### 3.5. ((3S*,6R*)-7,8,10-Trimethoxy-9-methyl-1,4-dioxo-3-(2,4,5-trimethoxy-3-methylbenzyl)-1,3,4,6-tetrahydro-2H-pyrazino(1,2-b)isoquinolin-6-yl)methyl Benzoate (**11a**) and ((3R*,6R*)-7,8,10-trimethoxy-9-methyl-1,4-dioxo-3-(2,4,5-trimethoxy-3-methylbenzyl)-1,3,4,6-tetrahydro-2H-pyrazino-(1,2-b)isoquinolin-6-yl)methyl Benzoate (**11b**)

A solution of **10** (644.0 mg, 1.0 mmol) in MeOH (50 mL) was hydrogenated over 10% Pd/C (213.0 mg) at 25 °C for 22 h. The catalyst was removed by filtration and washed with CHCl_3_ and MeOH. The combined filtrate was concentrated *in vacuo*. The residue was subjected to column chromatography with ethyl acetate–hexane (1:2) to give **11b** (137.0 mg, 21%) as a pale yellow amorphous powder, and with ethyl acetate–hexane (2:1) to give **11a** (463.0 mg, 72%) as a pale yellow amorphous powder.

Compound **11a**: ^1^H-NMR (400 MHz, CDCl_3_) δ: 8.00 (2H, m, Ar-H), 7.53 (1H, m, Ar-H), 7.42 (2H, m, Ar-H), 7.25 (1H, s, C11-H), 6.53 (1H, dd, *J* = 7.5, 4.2 Hz, C6-H), 6.51 (1H, s, C6′-H), 6.22 (1H, s, *N*-H), 4.45 (1H, dd, *J* = 11.4, 7.5 Hz, C12-H), 4.34 (1H, dd, *J* = 10.3, 3.3 Hz, C3-H), 4.31 (1H, dd, *J* =11.4, 4.2 Hz, C12-H), 3.97 (3H, s, C7-OMe), 3.82 (3H, s, C8-OMe), 3.80 (3H, s, C5′-OMe), 3.78 (3H, s, C4′-OMe), 3.67 (3H, s, C10-OMe), 3.64 (3H, s, C2′-OMe), 3.52 (1H, dd, *J* =13.8, 3.3 Hz, C3a-H), 2.73 (1H, dd, *J* =13.8, 10.3 Hz, C3a-H), 2.20 (3H, s, C3′-Me), 2.19 (3H, s, C9-Me). ^13^C-NMR (100 MHz, CDCl_3_) δ: 166.1 (s, OCOPh), 165.4 (s, C4), 160.7 (s, C1), 153.2 (s, C8), 151.8 (s, C10), 150.9 (s, C2′), 149.7 (s, C5′), 147.6 (s, C4′), 146.0 (s, C7), 133.1 (d, Ph), 129.9 (d, Ph × 2), 129.8 (s, Ph), 128.3 (d, Ph × 2), 126.4 (s, C9), 126.3 (s, C3′), 126.3 (s, C11a), 123.7 (s, C1′), 121.2 (s, C6a), 119.1 (s, C10a), 111.5 (d, C6′), 110.7 (d, C11), 64.6 (t, C12), 62.0 (q, C10-OCH_3_), 60.8 (q, C7-OCH_3_), 60.6 (q, C2′-OCH_3_), 60.2 (q, C4′-OCH_3_), 60.1 (q, C8-OCH_3_), 56.0 (q, C5′-OCH_3_) 55.3 (d, C3), 47.8 (d, C6), 33.5 (t, C3a), 9.7 (q, C3′-CH_3_), 9.4 (q, C9-CH_3_).

FT-IR (KBr) cm^−1^: 3391, 1724, 1690, 1628, 1489, 1468, 1458, 1414, 1368, 1337, 1269, 1240, 1121, 1088. EI-MS *m/z* (%): 646 (M^+^, 14), 511 (100), 509 (13), 483 (36), 260 (8), 232 (17), 195 (10). HR-EI-MS: calcd for C_35_H_38_N_2_O_10_, 646.2527, found: 646.2523. 

Compound **11b**: ^1^H-NMR (400 MHz, CDCl_3_) δ: 7.93 (2H, m, Ar-H), 7.52 (1H, m, Ar-H), 7.39 (2H, m, Ar-H), 6.91 (1H, s, C11-H), 6.43 (1H, dd, *J* = 7.1, 4.4 Hz, C6-H), 6.39 (1H, s, C6′-H), 4.45 (1H, dd, *J* = 8.9, 5.2 Hz, C3-H), 4.36 (1H, dd, *J* = 11.7, 7.1 Hz, C12-H), 4.31 (1H, dd, *J* =11.7, 4.4 Hz, C12-H), 3.98 (3H, s, C7-OMe), 3.83 (3H, s, C8-OMe), 3.66 (3H, s, C4′-OMe), 3.62 (3H, s, C5′-OMe), 3.61 (3H, s, C10-OMe), 3.57 (3H, s, C2′-OMe), 3.08 (1H, dd, *J* = 14.5, 8.9 Hz, C3a-H), 3.06 (1H, dd, *J* = 14.5, 5.2 Hz, C3a-H), 2.17 (3H, s, C9-Me), 2.05 (3H, s, C3′-Me). ^13^C-NMR (100 MHz, CDCl_3_) δ: 166.2 (s, OCOPh), 165.0 (s, C4), 160.6 (s, C1), 153.0 (s, C8), 151.5 (s, C10), 151.5 (s, C2′), 149.1 (s, C5′), 147.7 (s, C4′), 145.9 (s, C7), 133.0 (d, Ph), 129.7 (s, Ph), 129.7 (d, Ph × 2), 128.3 (d, Ph × 2), 126.2 (s, C9), 125.8 (s, C3′), 125.4 (s, C11a or C10a), 121.9 (s, C1′), 120.7 (s, C6a), 119.0 (s, C11a or C10a), 112.0 (d, C6′), 109.3 (d, C11), 64.9 (t, C12), 61.9 (q, C10-OCH_3_), 60.7 (q, C7-OCH_3_), 60.5 (q, C2′-OCH_3_), 60.2 (q, C4′-OCH_3_), 60.1 (q, C8-OCH_3_), 57.0 (d, C3), 55.8 (q, C5′-OCH_3_), 47.9 (d, C6), 36.5 (t, C3a), 9.2 (q, C9-CH_3_ and C3′-CH_3_). FT-IR (KBr) cm^−1^: 3300, 1724, 1692, 1628, 1487, 1468, 1414, 1379, 1321, 1271, 1119, 1088. EI-MS *m*/*z* (%): 646 (M^+^, 11), 511 (100), 483 (39), 232 (16), 195 (9). HR-EI-MS: calcd for C_35_H_38_N_2_O_10_, 646.2527, found: 646.2521.

### 3.6. Isopropyl ((3S*,6R*)-7,8,10-Trimethoxy-9-methyl-1,4-dioxo-3-(2,4,5-trimethoxy-3-methylbenzyl)-1,3,4,6-tetrahydro-2H-pyrazino(1,2-b)isoquinolin-6-yl)methyl Benzoate (**18**)

A solution of **11a** (16.47 g, 25 mmol), TEA (28.10 mL, 200 mmol), and DMAP (12.21 g, 100 mmol) in dichloromethane (400 mL) was cooled with ice water, and isopropyl chloroformate (40.08 mL, 350 mmol) was added dropwise over 30 min. The reaction mixture was stirred at 25 °C for 4 h. The organic layer was diluted with dichloromethane (500 mL), washed with 1 M aqueous HCl (500 mL ×2) and then water (500 mL), dried, and concentrated *in vacuo* to give a residue. The residue was subjected to column chromatography with ethyl acetate–hexane (2:5) to give **18** (17.5 g, 96%) as a yellow amorphous powder.

Compound **18**: ^1^H-NMR (400 MHz, CDCl_3_) δ: 8.00 (2H, m, Ar-H), 7.48 (1H, m, Ar-H), 7.34 (2H, m, Ar-H), 7.19 (1H, s, C11-H), 6.53 (1H, t, *J* = 3.4 Hz, C6-H), 5.24 (1H, t, *J* = 6.1 Hz, C3-H), 4.96 (1H, sept, *J* = 6.3 Hz, CO_2_CH(CH_3_)_2_), 4.65 (1H, dd, *J* = 11.5, 3.4 Hz, C12-H), 4.36 (1H, dd, *J* = 11.5, 3.4 Hz, C12-H), 3.92 (3H, s, C7-OMe), 3.80 (3H, s, C4′-OMe or C5′-OMe), 3.78 (3H, s, C8-OMe), 3.63 (3H, s, C4′-OMe or C5′-OMe), 3.59 (3H, s, C2′-OMe), 3.44 (3H, s, C10-OMe), 3.34 (1H, dd, *J* = 13.7, 6.1 Hz, C3a-H), 3.17 (1H, dd, *J* = 13.7, 6.1 Hz, C3a-H), 2.10 (3H, s, C9-Me), 2.09 (3H, s, C3′-Me), 1.28 (3H, d, *J* = 6.3 Hz, CO_2_CH(CH_3_)_2_), 1.20 (3H, d, *J* = 6.3 Hz, CO_2_CH(CH_3_)_2_). ^13^C-NMR (100 MHz, CDCl_3_) δ: 165.7 (s, OCOPh), 163.4 (s, C4), 157.6 (s, C1), 153.4 (s, C8), 151.7 (s, CO_2_CH(CH_3_)_2_), 151.6 (s, C10), 151.4 (s, C2′), 149.0 (s, C4′ or C5′), 147.4 (s, C4′ or C5′), 145.8 (s, C7), 133.0 (d, Ph), 129.7 (d, Ph × 2), 129.6 (s, Ph), 128.3 (d, Ph × 2), 126.8 (s, C11a or C10a), 126.2 (s, C9), 125.6 (s, C3′), 122.5 (s, C1′), 120.0 (s, C6a), 119.3 (s, C11a or C12), 112.0 (d, C6′), 111.6 (d, C11), 71.8 (d, CO_2_CH(CH_3_)_2_), 66.3 (t, C12), 61.8 (q, C10-OCH_3_), 60.6 (q, C2′-OCH_3_), 60.5 (q, C7-OCH_3_), 60.0 (q, C4′-OCH_3_ or C5′-OCH_3_), 60.0 (q, C8-OCH_3_), 59.4 (d, C3), 55.9 (q, C4′-OCH_3_ or C5′-OCH_3_), 49.9 (d, C6), 35.8 (t, C3a), 21.6 (q, CO_2_CH(CH_3_)_2_), 21.5 (q, CO_2_CH(CH_3_)_2_), 9.7 (q, C3′-CH_3_), 9.2 (q, C9-CH_3_). FT-IR (KBr) cm^−1^: 2938, 1722, 1684, 1468, 1418, 1391, 1375, 1344, 1271, 1252, 1107, 1092, 1072, 712. EI-MS *m*/*z* (%): 732 (M^+^, 12), 597 (100), 569 (19), 555 (6), 511 (8), 483 (21), 415 (9), 260 (13), 232 (19), 195 (13). HR-EI-MS: calcd for C_39_H_44_N_2_O_12_, 732.2894, found: 732.2889. 

### 3.7. Isopropyl (6S*,9R*,15R*)-9-((benzoyloxy)methyl)-1,2,4,10,11,13-hexamethoxy-3,12-dimethyl-7-oxo-6,7,9,15-tetrahydro-5H-6,15-epiminobenzo(4,5)azocino(1,2-b)isoquinoline-16-carboxylate (**19**) 

A stirred solution of **18** (402.3 mg, 0.55 mmol) in THF (36 mL) was cooled with ice water and lithium tri-*tert*-butoxyaluminohydride (1.12 g, 4.4 mmol) was added over 10 min. After continued stirring at 25 °C for 30 min, anhydrous Na_2_SO_4_ (2 g) was added and the reaction mixture was quenched with water. The reaction mixture was filtered through Celite pad and then, the filtrate was diluted with brine (200 mL) and extracted with CHCl_3_ (3 × 200 mL). The combined extracts were washed with brine (200 mL), dried, and concentrated *in vacuo* to give a residue, which was used in the next step without further purification. A solution of the residue as above in formic acid (36 mL) was stirred at 25 °C for 30 min. The reaction mixture was concentrated *in vacuo*, and the residue was diluted with saturated aqueous NaHCO_3_ solution (80 mL) and extracted with CHCl_3_ (3 × 80 mL). The combined extracts were washed with brine (80 mL), dried, and concentrated *in vacuo* to give a residue. The residue was subjected to column chromatography with ethyl acetate–hexane (1:3) to give **19** (322.7 mg, 82%) as a pale yellow amorphous powder.

Compound **19**: ^1^H-NMR (400 MHz, DMSO, 140 °C) δ: 7.58 (2H, m, Ar-H), 7.54 (1H, m, Ar-H), 7.41 (2H, m, Ar-H), 6.26 (1H, s, C14-H), 6.22 (1H, dd, *J* = 8.1, 4.2 Hz, C9-H), 5.93 (1H, d, *J* = 1.5, C15-H), 4.98 (1H, m, 1.5 Hz, C6-H), 4.86 (1H, sept, *J* = 6.2 Hz, CO_2_CH(CH_3_)), 3.92 (1H, dd, *J* = 11.5, 4.2 Hz, C16-H), 3.87 (3H, s, C2-OMe or C11-OMe), 3.87 (3H, s, C10-OMe), 3.85 (1H, dd, *J* = 11.5, 8.1 Hz, C16-H), 3.75 (3H, s, C4-OMe or C13-OMe), 3.73 (3H, s, C2-OMe or C11-OMe), 3.67 (3H, s, C4-OMe or C13-OMe), 3.51 (3H, s, C1-OMe), 3.09 (1H, br d, *J* =17.6 Hz, C5-H), 3.04 (1H, dd, *J* =17.6, 3.9 Hz, C5-H), 2.15 (3H, s, C3-Me or C12-Me), 1.98 (3H, s, C3-Me or C12-Me), 1.23 (3H, d, *J* = 6.2 Hz, CO_2_CH(CH_3_)_2_), 1.21 (3H, d, *J* = 6.2 Hz, CO_2_CH(CH_3_)_2_). ^13^C-NMR (100 MHz, DMSO, 140 °C) δ: 165.4 (s, C7), 164.4 (s, OCOPh), 152.1 (s, CO_2_CH(CH_3_)_2_), 151.7 (s, C1), 150.2 (s, C2 or C11), 149.2 (s, C4 and C13), 145.0 (s, C2 or C11), 144.7 (s, C10), 132.3 (s, C14a), 131.9 (d, Ph), 128.7 (s, Ph), 128.3 (d, Ph × 2), 127.5 (d, Ph × 2), 124.5 (s, C3 or C12), 124.0 (s, C3 or C12), 123.7 (s, C4a), 119.9 (s, C15a), 118.8 (s, C13a), 118.2 (s, C9a), 100.0 (d, C14), 68.8 (d, CO_2_CH(CH_3_)_2_), 62.6 (t, C16), 60.3 (q, C4-OCH_3_ or C13-OCH_3_), 59.8 (q, C2-OCH_3_ or C10-OCH_3_ or C11-OCH_3_), 59.2 (q, C2-OCH_3_ or C10-OCH_3_ or C11-OCH_3_), 59.1 (q, q, C2-OCH_3_ or C10-OCH_3_ or C11-OCH_3_), 58.9 (q, C4-OCH_3_ or C13-OCH_3_), 58.6 (q, C1-OCH_3_), 52.5 (d, C6), 48.9 (d, C15), 45.5 (d, C9), 26.9 (t, C5), 20.9 (q, CO_2_CH(CH_3_)_2_), 20.9 (q, CO_2_CH(CH_3_)_2_), 8.24 (q, C3-CH_3_ or C12-CH_3_), 8.18 (q, C3-CH_3_ or C12-CH_3_). FT-IR (KBr) cm^−1^: 1717, 1707, 1686, 1647, 1466, 1414, 1362, 1344, 1298, 1269, 1109, 1070, 1007, 964, 712. EI-MS *m/z* (%): 716 (M^+^, 22), 581 (100), 553 (67), 234 (21). HR-EI-MS: calcd for C_39_H_44_N_2_O_11_, 716.2945, found: 716.2942. 

### 3.8. ((6S*,9R*,15R*)-1,2,4,10,11,13-hexamethoxy-3,12-dimethyl-7-oxo-6,7,9,15-tetrahydro-5H-6,15-epiminobenzo(4,5)azocino(1,2-b)isoquinolin-9-yl)methyl Benzoate (**20**)

Concentrated H_2_SO_4_ (1.7 mL) was added to a stirred solution of **19** (322.7 mg, 0.45 mmol) in TFA (34 mL) at 0 °C over 5 min, and the reaction mixture was stirred at 25 °C for 4 h. The reaction mixture was poured into water (40 mL) at 0 °C, basified with concentrated NH_4_OH, and then extracted with CHCl_3_ (3 × 100 mL). The combined extracts were washed brine (100 mL), dried, and concentrated *in vacuo* to give a residue. The residue was subjected to column chromatography with ethyl acetate–hexane (1:3) to give **20** (267.6 mg, 94%) as a pale yellow amorphous powder.

Compound **20**: ^1^H-NMR (400 MHz, CDCl_3_) δ: 7.70 (2H, m, Ar-H), 7.49 (1H, m, Ar-H), 7.38 (2H, m, Ar-H), 6.41 (1H, dd, *J* = 8.1, 5.2 Hz, C9-H), 6.26 (1H, s, C14-H), 4.99 (1H, s, C15-H), 4.12 (1H, br d, *J* = 6.3 Hz, C6-H), 3.95~3.89 (2H, overlapped, C16-H), 3.90 (3H, s, C10-OMe), 3.87 (3H, s, C1-OMe), 3.76 (3H, s, C13-OMe), 3.76 (3H, s, C11-OMe), 3.67 (3H, s, C2-OMe), 3.44 (3H, s, C4-OMe), 3.19 (1H, dd, *J* = 17.0, 1.3 Hz, C5-H), 3.07 (1H, dd, *J* = 17.0, 6.3 Hz, C5-H), 2.19 (3H, s, C12-Me), 1.88 (3H, s, C3-Me). ^13^C-NMR (100 MHz, CDCl_3_) δ: 168.5 (s, C7), 166.0 (s, OCOPh), 152.5 (s, C4), 150.8 (s, C11), 149.9 (s, C2), 149.8 (s, C13), 146.3 (s, C1), 146.0 (s, C10), 136.1 (s, C13a or C14a), 132.5 (d, Ph), 129.6 (d, Ph), 129.4 (s, Ph), 128.3 (d, Ph), 125.7 (s, C12), 125.3 (s, C15a), 125.0 (s, C3), 121.5 (s, C4a), 120.4 (s, C13a or C14a), 119.5 (s, C9a), 99.8 (d, C14), 63.4 (t, C16), 61.4 (q, C13-OCH_3_), 60.8 (q, C10-OCH_3_), 60.2 (q, C1-OCH_3_), 60.1 (q, C11-OCH_3_), 59.9 (q, C2-OCH_3_), 59.3 (q, C4-OCH_3_), 53.9 (d, C6), 50.1 (d, C15), 45.7 (d, C9), 29.0 (t, C5), 9.3 (q, C12-CH_3_), 9.1 (q, C3-CH_3_). FT-IR (KBr) cm^−1^: 2938, 1722, 1680, 1638, 1466, 1412, 1362, 1271, 1248, 1115, 1070, 1009, 964, 712. EI-MS *m*/*z* (%): 630 (M^+^, 22), 495 (48), 467 (100), 234 (39), 204 (18). HR-EI-MS: calcd for C_35_H_38_N_2_O_9_, 630.2577, found: 630.2581. 

### 3.9. ((6S*,9R*,15R*)-1,2,4,10,11,13-hexamethoxy-3,12,16-trimethyl-7-oxo-6,7,9,15-tetrahydro-5H-6,15-epiminobenzo(4,5)azocino(1,2-b)isoquinolin-9-yl)methyl Benzoate (**21**)

A 37% aqueous solution of formaldehyde (6 mL) was added to a stirred solution of **20** (251.2 mg, 0.4 mmol) in formic acid (7.0 mL) at 60 °C, and the reaction mixture was heated at 70 °C for 1 h. The reaction mixture was diluted with 5% aqueous NaHCO_3_ solution (80 mL) and extracted with CHCl_3_ (3 × 80 mL). The combined extracts were washed brine (80 mL), dried, and concentrated *in vacuo* to give a residue. The residue was subjected to column chromatography with ethyl acetate–hexane (1:1) to give **21** (247.3 mg, 96%) as a colorless amorphous powder.

Compound **21**: ^1^H-NMR (400 MHz, CDCl_3_) δ: 7.71 (2H, m, Ar-H), 7.49 (1H, m, Ar-H), 7.38 (2H, m, Ar-H), 6.43 (1H, t, *J* = 6.4 Hz, C9-H), 6.30 (1H, s, C14-H), 4.63 (1H, s, C15-H), 3.92 (3H, s, C10-OMe), 3.91 (2H, d, *J* = 6.4 Hz, C17-H), 3.87 (3H, s, C1-OMe), 3.76 (6H, s, C11-OMe and C13-OMe), 3.68 (1H, overlapped, C6-H), 3.67 (3H, s, C2-OMe), 3.43 (3H, s, C4-OMe), 3.15 (1H, dd, *J* = 17.6, 4.1 Hz, C5-H), 3.11 (1H, br d, *J* = 17.6 Hz, C5-H), 2.55 (3H, s, *N*-Me), 2.20 (3H, s, C12-Me), 1.89 (3H, s, C3-Me). ^13^C-NMR (100 MHz, CDCl_3_) δ: 168.1 (s, C7), 166.0 (s, OCOPh), 152.4 (s, C4), 150.7 (s, C11), 149.8 (s, C2), 149.5 (s, C13), 146.1 (s, C1), 145.9 (s, C10), 132.7 (s, C14a), 132.5 (d, Ph), 129.6 (d, Ph × 2), 129.3 (s, Ph), 128.3 (d, Ph × 2), 126.0 (s, C15a), 125.7 (s, C12), 124.7 (s, C3), 121.1 (s, C4a), 119.9 (s, C13a), 119.6 (s, C9a), 102.7 (d, C14), 63.6 (t, C17), 61.3 (q, C11-OCH_3_ or C13-OCH_3_), 60.7 (q, C10-OCH_3_), 60.5 (d, C6), 60.1 (q, C1-OCH_3_), 60.0 (q, C11-OCH_3_ or C13-OCH_3_), 59.8 (q, C2-OCH_3_), 59.2 (q, C4-OCH_3_), 56.8 (d, C15), 45.5 (d, C9), 41.6 (q, *N*-CH_3_), 29.2 (t, C5), 9.2 (q, C12-CH_3_), 9.0 (q, C3-CH_3_). FT-IR (KBr) cm^−1^: 1722, 1678, 1638, 1464, 1412, 1358, 1341, 1271, 1126, 1113, 1099, 1067, 1007, 712. EI-MS *m/z* (%): 644 (M^+^, 17), 509 (20), 481 (100), 248 (37), 218 (15). HR-EI-MS: calcd for C_36_H_40_N_2_O_9_, 644.2734, found: 644.2733. 

### 3.10. (6S*,9R*,15R*)-9-(hydroxymethyl)-1,2,4,10,11,13-hexamethoxy-3,12,16-trimethyl-5,6,9,15-tetrahydro-7H-6,15-epiminobenzo(4,5)azocino(1,2-b)isoquinolin-7-one (**12**)

A 10 M aqueous solution of lithium hydroxide monohydrate (77 μL, 0.77 mmol) was added to a stirred solution of **21** (226.3 mg, 0.35 mmol) in THF (2.0 mL) and MeOH (0.7 mL), and stirring was continued at 25 °C for 8 h. The reaction mixture was diluted with water (80 mL), and the mixture was extracted with CHCl_3_ (3 × 80 mL). The combined extracts were washed with brine (80 mL), dried, and concentrated *in vacuo* to give a residue. The residue was subjected to column chromatography with ethyl acetate–hexane (2:1) to give **12** (184.1 mg, 97%) as a colorless amorphous powder. 

Compound **12**: ^1^H-NMR (400 MHz, CDCl_3_) δ: 6.27 (1H, s, C14-H), 6.08 (1H, dd, *J* = 7.9, 5.1 Hz, C9-H), 4.70 (1H, brs, C15-H), 3.88 (3H, s, OMe), 3.86 (3H, s, OMe), 3.78 (3H, s, OMe), 3.77 (3H, s, OMe), 3.75 (3H, s, OMe) 3.72 (1H, br t, C6-H), 3.66 (3H, s, OMe), 3.32 (1H, dt, *J* = 11.5, 5.1 Hz, C17-H), 3.22 (1H, dt, *J* = 11.5, 7.9 Hz, C17-H), 3.21 (2H, d, *J* = 4.4 Hz, C5-H), 2.57 (3H, s, *N*-Me), 2.19 (3H, s, C12-Me), 2.18 (3H, s, C3-Me), 1.37 (1H, t, *J* = 6.1 Hz, -OH). ^13^C-NMR (100 MHz, CDCl_3_) δ: 169.1 (C7), 152.6 (C4), 150.7 (C11), 150.1 (C2), 149.7 (C13), 146.3 (C1), 145.8 (C10), 132.8 (C14a), 126.0 (C15a), 125.4 (C12), 125.1 (C3), 121.1 (C4a), 120.6 (C9a), 119.5 (C13a), 102.9 (14), 64.6 (C17), 61.3 (-OCH_3_), 60.6 (-OCH_3_), 60.6 (C6), 60.1 (-OCH_3_), 60.1 (-OCH_3_), 60.0 (-OCH_3_), 59.8 (-OCH_3_), 56.5 (C15), 49.1 (C9), 41.6 (*N*-CH_3_), 29.3 (C5), 9.4 (-CH_3_), 9.2 (-CH_3_). FT-IR (KBr) cm^−1^: 3468, 2940, 1672, 1636, 1466, 1412, 1341, 1248, 1113, 1065, 1007, 964. EI-MS *m/z* (%): 540 (M^+^, 9), 509 (27), 481 (100), 248 (51). HR-EI-MS: calcd for C_29_H_36_N_2_O_8_, 540.2472, found: 540.2473. 

### 3.11. ((6S*,9R*,15R*)-1,2,4,10,11,13-hexamethoxy-3,12,16-trimethyl-7-oxo-6,7,9,15-tetrahydro-5H-6,15-epiminobenzo(4,5)azocino(1,2-b)isoquinolin-9-yl)methyl Benzoate (**22**)

To a stirred solution of **12** (216.0 mg, 0.4 mmol) in CH_2_Cl_2_ (24 mL) at −78 °C was added a CH_2_Cl_2_ solution of BBr_3_ (1.0 M, 2.40 mL, 2.4 mmol) over 5 min. Stirring was continued at the same temperature for 1 h, and then at −20 °C for 14.5 h. The reaction mixture was diluted with water (200 mL) and extracted with 5% MeOH in CHCl_3_ (4 × 200 mL). The combined extracts were washed with 5% NaHCO_3_ solution (200 mL), dried, and concentrated *in vacuo* to give a residue. A solution of the above residue in 10 N HNO_3_ (5.0 mL) was stirred at 25 °C for 30 min. The reaction mixture was diluted with water (150 mL) and extracted with ethyl acetate (3 × 200 mL). The combined extracts were washed with brine (200 mL), dried, and concentrated *in vacuo*. The residue was subjected to purification by silica gel chromatography with ethyl acetate to give **22** (130.0 mg, 67%) as a dark purple amorphous powder. 

Compound **22**: ^1^H-NMR (500 MHz, CDCl_3_) δ: 6.26 (1H, s, 14-H), 5.96 (1H, dd, *J* = 7.1, 4.5 Hz, 9-H), 4.55 (1H, s, 15-H), 4.02 (3H, s, OCH_3_), 3.97 (3H, s, OCH_3_), 3.74 (1H, dt, *J* = 6.5, 1.5 Hz, 6-H), 3.50 (1H, dd, *J* = 11.4, 4.5 Hz, 17-H), 3.36 (1H, dd, *J* = 11.4, 7.1 Hz, 17-H), 2.96 (1H, dd, *J* = 19.8, 6.5 Hz, 5-Hα), 2.89 (1H, dd, *J* = 19.8, 1.5 Hz, C5-Hβ), 2.51 (3H, s, *N*-CH_3_), 1.96 (3H, s, 3-CH_3_ or 12-CH_3_), 1.94 (3H, s, 3-CH_3_ or 12-CH_3_), 1.62 (1H, br s, OH). ^13^C-NMR (125 MHz, CDCl_3_) δ: 186.6 (s, C-4), 185.0 (s, C-13), 180.5 (s, C-10 and C-1), 168.0 (s, C-7), 156.0 (s, C-11), 155.4 (s, C-2), 140.7 (s, C-14a), 140.0 (s, C-4a), 136.5 (s, C-15a), 134.5 (s, C-13a), 129.1 (s, C-3), 127.6 (s, C-12), 125.0 (s, C-9a), 101.8 (d, C-14), 62.9 (t, C-17), 61.1 (q, OCH_3_), 61.0 (q, OCH_3_), 59.6 (d, C-6), 54.4 (d, C-15), 48.4 (d, C-9), 41.2 (q, *N*-CH_3_), 28.7 (t, C-5), 8.8 (q, Ar-CH_3_), 8.7 (q, Ar-CH_3_). FT-IR (KBr) cm^−1^: 3347, 2951, 2855, 1654, 1616, 1568, 1450, 1373, 1310. LR-MS (FAB^+^): 481 [M + H]^+^. HR-MS (FAB^+^): calcd for C_25_H_25_N_2_O_8_, 481.1611, found: 481.1623.

### 3.12. ((6S*,9R*,15R*)-2,11-dimethoxy-3,12,16-trimethyl-1,4,7,10,13-pentaoxo-1,5,6,7,9,10,13,15-octa-hydro-4H-6,15-epiminobenzo(4,5)azocino(1,2-b)isoquinolin-9-yl)methyl (Z)-2-methylbut-2-enoate (**23**)

A solution of angelic acid (601.0 mg, 6.0 mmol) in ether (30 mL) was cooled with ice water, and a solution of oxalyl chloride (0.5 mL, 5.9 mmol) in DMF (46.0 μL, 592 mmol) was added dropwise over 5 min. The resulting solution was stirred at 25 °C for 2 h. Then, a solution of **22** (142.0 mg, 0.3 mmol) in CH_2_Cl_2_ (15 mL) was added over 5 min. The reaction mixture was concentrated to approximately 3.0 mL with a stream of argon, and CH_2_Cl_2_ (8.0 mL) was added. The resulting mixture was stirred at 25 °C for 21 h. The reaction mixture was directly purified by silica gel chromatography with ethyl acetate–hexane (2:1) to afford **23** (139.0 mg, 84%) as a dark purple film. 

Compound **23**: ^1^H-NMR (500 MHz, CDCl_3_) δ: 6.24 (1H, s, 14-H), 6.12 (1H, dd, *J* = 5.7, 2.9 Hz, 9-H), 5.92 (1H, qq, *J* = 7.4, 1.4 Hz, 21-H), 4.50 (1H, br s, 15-H), 4.21 (1H, dd, *J* = 11.9, 5.7 Hz, 17-H), 4.05 (3H, s, C11-OCH_3_), 4.01 (3H, s, 2-OCH_3_), 4.01 (1H, dd, *J* = 11.9, 2.9 Hz, 17-H), 3.72 (1H, dt, *J* = 6.8, 1.4 Hz, 6-H), 2.95 (1H, dd, *J* = 19.8, 6.8 Hz, 5-Hβ), 2.84 (1H, dd, *J* = 19.8, 1.4 Hz, 5-H), 2.47 (3H, s, *N*-CH_3_), 1.96 (3H, s, 12-CH_3_), 1.92 (3H, s, 3-CH_3_), 1.75 (3H, dq, *J* = 7.4, 1.4 Hz, 21-CH_3_), 1.57 (1H, quint, *J* = 1.4 Hz, 20-CH_3_). ^13^C-NMR (125 MHz, CDCl_3_) δ: 186.5 (s, C-4), 184.9 (s, C-13), 180.5 (s, C-1), 180.1 (s, C-10), 167.1 (s, C-7 and C-19), 156.2 (s, C-11), 155.2 (s, C-2), 140.6 (s, C-14a), 139.8 (s, C-4a), 139.3 (d, C-21), 136.2 (s, C-15a), 134.6 (s, C-13a), 128.5 (s, C-3), 127.3 (s, C-12), 126.8 (s, C-20), 124.2 (s, C-9a), 101.3 (d, C-14), 62.4 (t, C-17), 61.1 (q, 11-OCH_3_), 61.0 (q, 2-OCH_3_), 59.5 (d, C-6), 54.3 (d, C-15), 47.1 (d, C-9), 41.1 (q, *N*-CH_3_), 28.3 (t, C-5), 20.2 (q, C-23), 15.5 (q, C-22), 8.7 (q, 3-CH_3_), 8.6 (q, 12-CH_3_). FT-IR (KBr) cm^−1^: 2949, 1653, 1616, 1570, 1458, 1309, 1228, 1153. EI-MS *m*/*z* (%): 562 (M^+^, 5), 421 (100), 218 (40). HR-EI-MS: calcd for C_30_H_30_N_2_O_9_, 562.1951, found: 562.1952. 

### 3.13. Renieramycin I (**1i**)

A suspension of **23** (15.0 mg, 0.027 mmol) and SeO_2_ (29.6 mg, 0.27 mmol) in dioxane (3.0 mL) and MeOH (1.0 mL) was heated at 100 °C for 6 days. The reaction mixture was filtered and the filter cake was washed with ethyl acetate. The combined filtrates were concentrated *in vacuo* to give a residue. Flash column chromatography on silica gel with ethyl acetate–hexane (2:3) afforded **1i** (6.8 mg, 43%) as a dark red film and **24** (4.4 mg, 29%).

Renieramycin I (**1i**): ^1^H-NMR (400 MHz, CDCl_3_) δ: 6.26 (1H, s, 4-H), 6.07 (1H, dd, *J* = 5.5, 2.7 Hz, 1-H), 5.92 (1H, qq, *J* = 7.3, 1.4 Hz, 26-H), 4.54 (1H, d, *J* = 0.9 Hz, 11-H), 4.34 (1H, d, *J* = 1.4 Hz, 14-H), 4.16 (1H, dd, *J* = 12.1, 5.5 Hz, 22-H), 4.06 (3H, s, 7-OCH_3_), 4.02 (1H, dd, *J* = 12.1, 2.7 Hz, 22-H), 3.98 (3H, s, 17-OCH_3_), 3.74 (1H, br t, *J* = 1.4 Hz, 13-H), 3.62 (3H, s, 14-OCH_3_), 2.55 (3H, s, *N*-CH_3_), 1.96 (3H, s, 6-CH_3_), 1.94 (3H, s, 16-CH_3_), 1.73 (3H, dq, *J* = 7.3, 1.4 Hz, 26-CH_3_), 1.55 (3H, quint, *J* = 1.4 Hz, 25-CH_3_). ^13^C-NMR (100 MHz, CDCl_3_) δ: 185.8 (s, C-15), 184.7 (s, C-5), 180.9 (s, C-18), 180.1 (s, C-8), 167.1 (s, C-24), 164.1 (s, C-21), 156.1 (s, C-7), 155.2 (s, C-17), 139.2 (d, C-26), 138.6 (s, C-3), 137.4 (s, C-19), 137.0 (s, C-20), 134.2 (s, C-10), 129.3 (s, C-16), 127.4 (s, C-6), 126.7 (s, C-25), 124.9 (s, C-9), 102.2 (d, C-4), 73.4 (d, C-14), 64.7 (d, C-13), 62.6 (t, C-22), 61.2 (q, 7-OCH_3_), 60.9 (q, 17-OCH_3_), 59.4 (q, 14-OCH_3_), 54.5 (d, C-11), 47.3 (d, C-1), 41.7 (q, *N*-CH_3_), 20.2 (q, 25-CH_3_), 15.5 (q, 26-CH_3_), 8.9 (q, Ar-CH_3_), 8.7 (q, Ar-CH_3_). FT-IR (KBr) cm^−1^: 3429, 2949, 1717, 1684, 1655, 1614, 1570, 1454, 1342, 1306, 1233, 1209, 1153, 1096. EI-MS *m*/*z* (%): 594 ([M + 2H]^+^, 0.7), 593 ([M + H]^+^, 2), 592 (M^+^, 6), 479 (19), 452 (26), 451 (100), 421 (20), 248 (25), 218 (11). HR-EI-MS: calcd for C_30_H_30_N_2_O_10_, 592.2057, found: 592.2056.

### 3.14. ((6S*,9R*,15R*)-5-hydroxy-2,11-dimethoxy-3,12,16-trimethyl-1,4,7,10,13-pentaoxo-1,5,6,7,9,10,13,15-octahydro-4H-6,15-epiminobenzo(4,5)azocino(1,2-b)isoquinolin-9-yl)methyl (Z)-2-methylbut-2-enoate (**24**) 

^1^H-NMR (500 MHz, CDCl_3_) δ: 6.28 (1H, s, 4-H), 6.09 (1H, dd, *J* = 6.0, 2.9 Hz, 1-H), 5.93 (1H, qq, *J* = 7.3, 1.5 Hz, 26-H), 4.86 (1H, br d, *J* = 7.0 Hz, 14-H), 4.52 (1H, d, *J* = 1.1 Hz, 11-H), 4.19 (1H, dd, *J* = 12.0, 6.0 Hz, 22-H), 4.06 (3H, s, 7-OCH_3_), 4.01 (3H, s, 17-OCH_3_), 3.99 (1H, dd, *J* = 12.0, 2.9 Hz, 22-H), 3.75 (1H, dd, *J* = 1.7, 1.1 Hz, 13-H), 2.88 (1H, br d, *J* = 7.0 Hz, OH), 2.55 (3H, s, *N*-CH_3_), 1.96 (3H, s, 6-CH_3_), 1.93 (3H, s, 16-CH_3_), 1.75 (3H, dq, *J* = 7.3, 1.5 Hz, 26-CH_3_), 1.56 (3H, quint, *J* = 1.5 Hz, 25-CH_3_). ^13^C-NMR (125 MHz, CDCl_3_) δ: 186.7 (s, C-15), 184.7 (s, C-5), 181.0 (s, C-18), 180.0 (s, C-8), 167.1 (s, C-24), 163.9 (s, C-21), 156.1 (s, C-7), 155.4 (s, C-17), 139.3 (d, C-26), 138.4 (s, C-10 or C-20), 138.3 (s, C-10 or C-20), 136.9 (s, C-19), 134.3 (s, C-3), 128.8 (s, C-16), 127.4 (s, C-6), 126.7 (s, C-25), 124.8 (s, C-9), 102.2 (d, C-4), 67.1 (d, C-13), 64.8 (d, C-14), 62.3 (t, C-22), 61.1 (q, OCH_3_), 61.0 (q, OCH_3_), 54.7 (d, C-11), 47.2 (d, C-1), 41.5 (q, *N*-CH_3_), 20.2 (q, 25-CH_3_), 15.5 (q, 26-CH_3_), 8.7 (q, Ar-CH_3_), 8.7 (q, Ar-CH_3_). FT-IR (KBr) cm^−1^: 3446, 2930, 2857, 1654, 1616, 1570, 1456, 1307, 1233, 1211, 1153. EI-MS *m*/*z* (%): 578 (M^+^, 5), 437 (100), 421 (48), 218 (20). HR-EI-MS: calcd for C_30_H_30_N_2_O_10_, 578.1900, found: 578.1899.

### 3.15. Cribrostatin 4 (**2**) via **24**

A suspension of **23** (112.4 mg, 0.2 mmol) and SeO_2_ (110.9 mg, 1.0 mmol) in dioxane (30 mL) and water (3.0 mL) was heated at 80 °C for 6 h. The reaction mixture was filtered and the filter cake was washed with ethyl acetate (200 mL). The combined filtrates were concentrated *in vacuo* to give a residue (248.3 mg). Flash column chromatography on silica gel (70 g) with hexane–ethyl acetate (1:1) afforded **24** (72.5 mg, 63%) along with recovered **23** (16.2 mg, 14%). Dess-Martin periodinane (DMP, 445.3 mg, 1.05 mmol) was added to a stirred solution of **24** (57.8 mg, 0.1 mmol) in dichloromethane (15 mL), and the mixture was stirred at 25 °C for 3 h. The reaction mixture was diluted with THF (50 mL), saturated aqueous Na_2_S_2_O_3_ solution (50 mL) was added, and the mixture was stirred at 25 °C for 2 h. The reaction mixture was diluted with water (100 mL) and extracted with ethyl acetate (100 mL × 3). The combined extracts were washed with brine (100 mL), dried, and concentrated *in vacuo*. The residue (230 mg) was purified by silica gel (9 g) flash column chromatography with hexane–ethyl acetate (1:2) to give cribrostatin 4 (**2**: 81.0 mg, 70.0% from **23**) as a dark red film. 1H-, 13C NMR, and also IR spectral charts of synthetic renieramycin **I** and cribrostatin **4** are available in the supplementary information.

## 4. Conclusions 

In summary, we have succeeded in reducing the number of steps in our first version of the total synthesis of cribrostatin 4 through key intermediate **12**, from 21 steps in 3.4% overall yield to 18 steps in 8.3% overall yield. The main point of this alternative total synthesis is based on the Avendano’s protocol introducing C3-C4 double bond in the early stage. We have completed the first total synthesis of renieramycin I (**1i**), and the spectroscopic data provide unambiguous evidence that supports the original structure of the natural product. The development of ways to utilize this approach for the synthesis of other members of the C3-C4 unsaturated renieramycin family, and the examination of their biological activities to evaluate the mechanism of action, are undergoing in our laboratory.

## Supplementary Files

Supplementary File 1
