# Supplementary material for: Chemistry of Renieramycins. Part 14: Total Synthesis of Renieramycin I and Practical Synthesis of Cribrostatin 4 (Renieramycin H)
Source: Mar Drugs. 2015 Aug 6;13(8):4915–33. doi: 10.3390/md13084915 (PMC4557007; doi:10.3390/md13084915)
Supplement: Supplementary File 1 [file marinedrugs-13-04915-s001.docx]

Supplementary Information

**Figure S1.** ^1^H NMR spectrum of Renieramycin **I**.

**Figure S2.** ^13^C NMR spectrum of Renieramycin **I**.


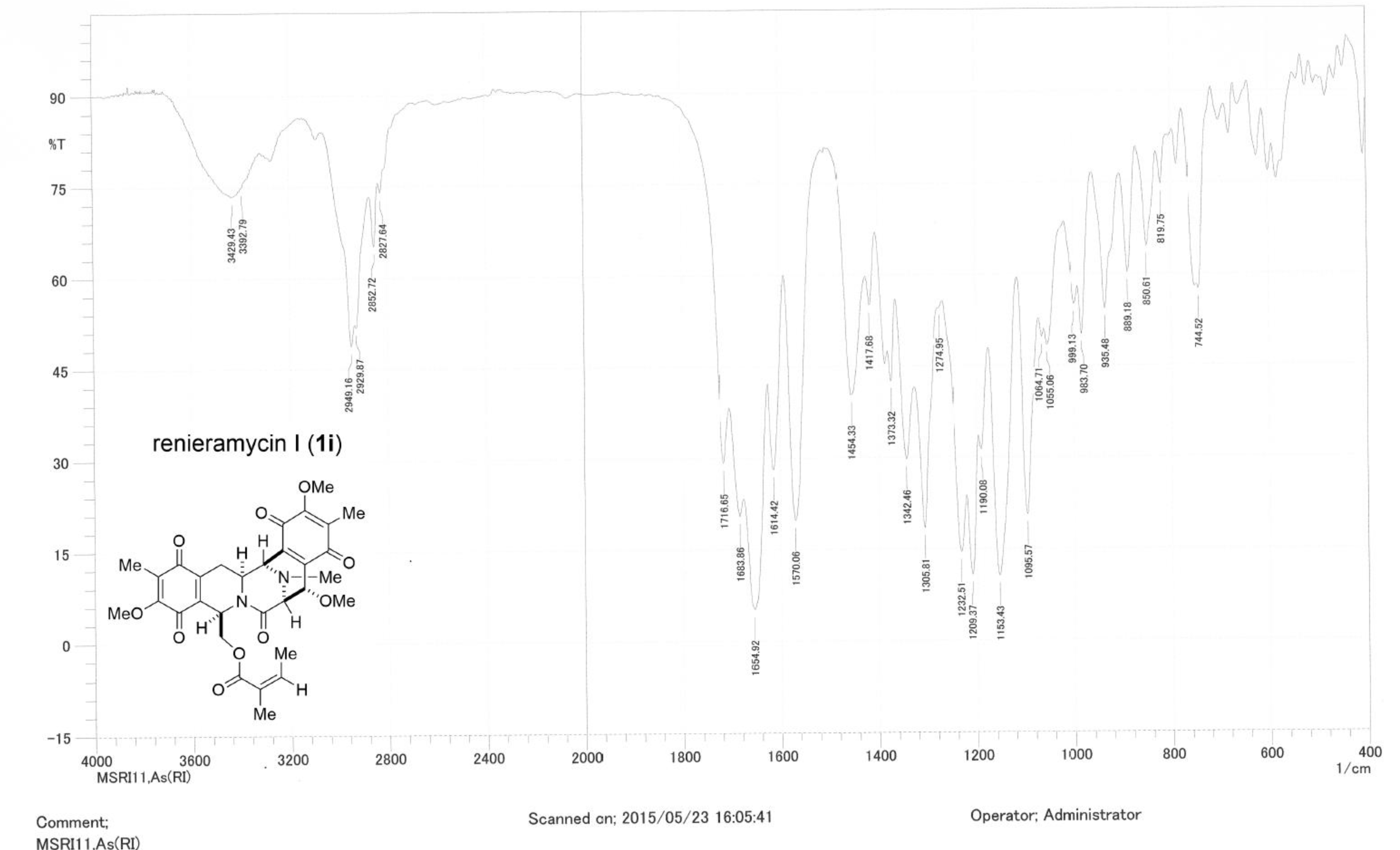


**Figure S3.** IR spectral of Renieramycin **I**.

**Figure S4.** ^1^H NMR spectrum of Cribrostatin **4**.

**Figure S5.** ^13^C NMR spectrum of Cribrostatin **4**.

**Figure S6.** IR spectral of Cribrostatin **4**.

© 2015 by the authors; licensee MDPI, Basel, Switzerland. This article is an open access article distributed under the terms and conditions of the Creative Commons Attribution license (http://creativecommons.org/licenses/by/4.0/).
